# Two-Drug Antimicrobial Chemotherapy: A Mathematical Model and Experiments with *Mycobacterium marinum*

**DOI:** 10.1371/journal.ppat.1002487

**Published:** 2012-01-12

**Authors:** Peter Ankomah, Bruce R. Levin

**Affiliations:** Department of Biology, Emory University, Atlanta, Georgia, United States of America; Harvard School of Public Health, United States of America

## Abstract

Multi-drug therapy is the standard-of-care treatment for tuberculosis. Despite this, virtually all studies of the pharmacodynamics (PD) of mycobacterial drugs employed for the design of treatment protocols are restricted to single agents. In this report, mathematical models and *in vitro* experiments with *Mycobacterium marinum* and five antimycobacterial drugs are used to quantitatively evaluate the pharmaco-, population and evolutionary dynamics of two-drug antimicrobial chemotherapy regimes. Time kill experiments with single and pairs of antibiotics are used to estimate the parameters and evaluate the fit of Hill-function-based PD models. While Hill functions provide excellent fits for the PD of each single antibiotic studied, rifampin, amikacin, clarithromycin, streptomycin and moxifloxacin, two-drug Hill functions with a unique interaction parameter cannot account for the PD of any of the 10 pairs of these drugs. If we assume two antibiotic-concentration dependent functions for the interaction parameter, one for sub-MIC and one for supra-MIC drug concentrations, the modified biphasic Hill function provides a reasonably good fit for the PD of all 10 pairs of antibiotics studied. Monte Carlo simulations of antibiotic treatment based on the experimentally-determined PD functions are used to evaluate the potential microbiological efficacy (rate of clearance) and evolutionary consequences (likelihood of generating multi-drug resistance) of these different drug combinations as well as their sensitivity to different forms of non-adherence to therapy. These two-drug treatment simulations predict varying outcomes for the different pairs of antibiotics with respect to the aforementioned measures of efficacy. In summary, Hill functions with biphasic drug-drug interaction terms provide accurate analogs for the PD of pairs of antibiotics and *M. marinum*. The models, experimental protocols and computer simulations used in this study can be applied to evaluate the potential microbiological and evolutionary efficacy of two-drug therapy for any bactericidal antibiotics and bacteria that can be cultured *in vitro.*

## Author Summary

The goal of this investigation is the development and *a priori* evaluation of multi-drug treatment regimes that are effective in clearing long-term bacterial infections like tuberculosis, and also minimize the likelihood of multi-drug resistance arising during therapy. To achieve this end, we use mathematical models and *in vitro* experiments with *Mycobacterium marinum* (a close relative of *M. tuberculosis*) and five different antimycobacterial agents to develop and validate realistic analogues of the pharmacodynamics of two-drug chemotherapy. All ten drug pairs examined exhibited the same general biphasic drug-drug interaction properties: at low concentrations (subMICs), the two drugs together were less effective than anticipated from their independent pharmacodynamics (were antagonistic), but as their concentrations increased, the interactions between the antibiotics became relatively more synergistic. Using computer simulations with these empirically estimated two-drug pharmacodynamic functions, we evaluated the relative efficacy of the different antibiotic combinations in terms of the anticipated rate of clearance of infections and the likelihood of resistance arising with and without non-adherence to a treatment regime. The simulations predict different outcomes for each of the drug combinations. The models and experimental methods used in this study can be applied to characterize any combinations of bactericidal antibiotics and evaluate their potential efficacy.

## Introduction

The concurrent use of multiple drugs, which is one of the mainstays of chemotherapy, is useful and in some cases necessary for the successful treatment of diseases such as tuberculosis (TB), HIV/AIDS, malaria and various cancers. Shortly after antimycobacterial agents became available for treating TB, it was recognized that single drug therapy almost invariably led to treatment failure due to the ascent of resistance, but that this could be mitigated by the use of multiple drugs with different modes of action [1]–[4]. In its current form, standard tuberculosis treatment consists of a two-month combinatorial course of rifampin, isoniazid, pyrazinamide and ethambutol, followed by a four-month continuation phase of isoniazid and rifampin.

Despite the barrage of antibiotics and long term of combination therapy, *Mycobacterium tuberculosis* (Mtb) strains that are resistant to multiple drugs are an increasingly troubling component of the epidemiological landscape. In 2009, the World Health Organization estimated close to half a million cases of multidrug resistant (MDR) TB (cases in which recovered strains were resistant to the most potent first-line antibiotics, rifampin and isoniazid) [5]. By mid-2010, 58 countries had reported at least one case of extensively drug-resistant (XDR) TB (MDR strains that are additionally resistant to any fluoroquinolone as well as at least one of the injectable drugs capreomycin, kanamycin and amikacin) [5]. The important issue is thus: how can the term of tuberculosis chemotherapy and the likelihood of treatment failure due to the evolution of resistance during the course of therapy be reduced?

One approach to improving the efficacy of single drug therapy has been to design treatment regimes based on *in vivo* data of the changes in the concentration of the antibiotic, pharmacokinetics (PK), and *in vitro* data on the relationship between the concentration of the drug and the rate of growth/death of the bacteria, pharmacodynamics (PD) [6]–[9]. This PK/PD approach to the rational design of antibiotic treatment regimes has been employed for tuberculosis but almost exclusively for single antibiotics [10]–[19]. To extend this approach to the multi-drug treatment regimes clearly needed to prevent acquired resistance, it is necessary to concurrently account for the PD of the different drugs, and most critically, how they interact [20]–[22].

Drug interactions are generally classified as antagonistic, synergistic or additive. In the case of bactericidal antibiotics, additive interactions are usually described in one of two ways, ‘Bliss Independence’ and ‘Loewe Additivity’. Bliss Independence asserts that each drug in a combination exerts its killing action independently of the other drugs [23]. For example, if there are two drugs, A and B, and at particular concentrations they kill f_a_ and f_b_ (0<f_a_,f_b_<1) fractions of a bacterial population in an hour, at the end of the hour the viable cell density would be reduced to (1-f_a_)(1-f_b_) of its initial level. For Loewe additivity, the fraction of surviving cells with both drugs would be 1-f_a_-f_b_, the constraint being that f_a_+f_b_<1 [24]. Antagonism and synergism can then be defined relative to one of these descriptions of additivity: drugs interact antagonistically if their combined cidal activity is less than would be predicted for an additive drug combination, and synergistically if the cidal activity is more.

Unfortunately, these definitions cannot be readily translated into the PD of two drugs as they do not account for how the rate or extent of killing would vary with the concentrations of the drug. To address this, Greco and colleagues proposed a seminal Emax-based two-drug pharmacodynamic function which assumes that a single parameter can account for the interaction between both drugs [25], [26]. If the value of this parameter is zero, then the drugs are additive, with a negative value indicating antagonism and a positive value indicating synergy. Although this and other Emax-based models have been used to characterize the nature of the interactions between different kinds of drugs, including antimicrobials [27]–[33], there has been limited quantitative consideration of how two-drug PD models apply to the design and evaluation of antibiotic treatment regimes for bacteria, particularly those, like tuberculosis, where multiple drug therapy is essential [27], [33].

In this study, we explore the fit of Hill functions (which subsume Emax models) for the PD of the antimycobacterial antibiotics rifampin, amikacin, clarithromycin, streptomycin and moxifloxacin. We then employ a Hill-function-based variant of the Greco model to explore the PD of the 10 possible pairs of these drugs. As our experimental organism, we use *Mycobacterium marinum*. In addition to being safer and more convenient to work with, *M. marinum* is a close genetic relative and shares numerous virulence determinants with Mtb. It also recapitulates key immunopathological features of human tuberculosis infection in its natural poikilothermic hosts [34]–[36].

To explore the potential clinical implications of these theoretical and *in vitro* PD studies, we use Monte Carlo simulations of antibiotic treatment and resistance that incorporate PD functions that best fit our data. Of particular concern in this analysis are: (i) the relative rates at which these different drug combinations clear the simulated infections (their microbiological efficacy) (ii) the likelihood of resistance to the two drugs evolving during the course of therapy (their evolutionary efficacy), and (iii) how that efficacy is affected by different forms of non-adherence to the treatment regime.

## Results

### Single drug pharmacodynamics

**Figure 1:**
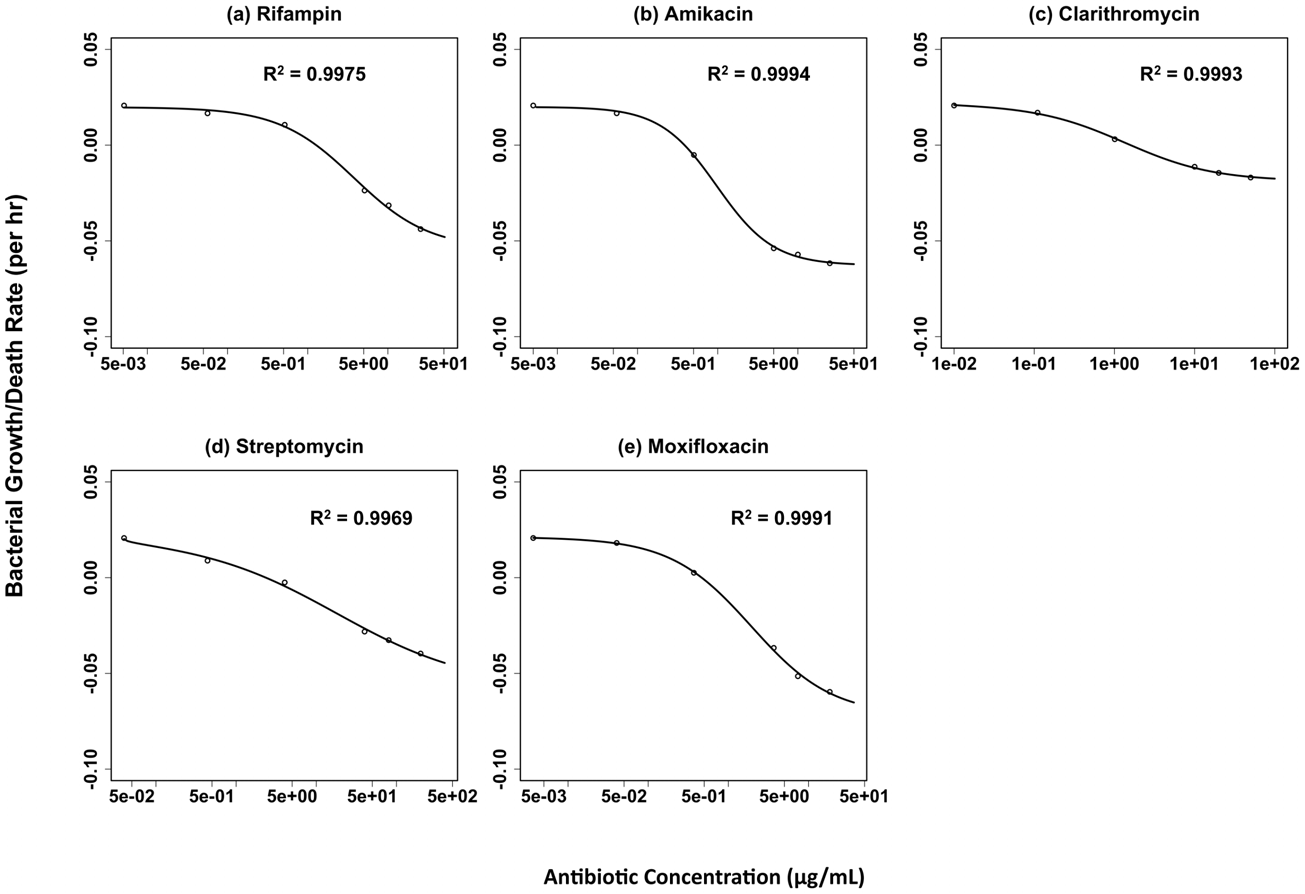
Fit of the Hill function to time-kill data for single antibiotics. Adjusted R^2^ values determined from an F test are shown. (a) Rifampin, (b) Amikacin, (c) Clarithromycin, (d) Streptomycin, (e) Moxifloxacin.

In Figure 1 we show the fit of the theoretical single-drug pharmacodynamic function (Equation 1) to the PD data obtained from experiments with five antimycobacterial agents. These data were generated by exposing *M. marinum* to the antibiotics at different concentrations and estimating net bacterial growth/death rates (based on the increase or decrease in the density of viable bacteria) over 72 hours. The analyses of these time-kill data were restricted to 72 hours in order to ensure that bacteria were growing and/or being killed exponentially.

For single antibiotics, the Hill function provides a good fit for the relationship between the concentration of the drug and the growth/death rate of the bacteria (Figure 1, see R^2^ values). This is also evident in Table 1, where we list the estimates of the Hill function parameters for each of the drugs. The maximum growth rates calculated from this function are very close to that estimated independently (data not shown). Moreover, the estimated zMIC's (MIC's calculated from the Hill functions) and MIC's determined by the CLSI [37] recommended broth dilution method are, given the factor of two limitation of the latter, coincident. The individual antibiotics exhibited different pharmacodynamic signatures reflected in the varying shapes of the PD function (the parameter κ) and the kill rate parameter ψ_min_, which ranged from −0.043 to −0.166 h^−1^.

**Table 1:** Single-drug pharmacodynamic function parameter estimates and standard errors.

Drug	ψ_max_ (h^−1^)	ψ_min_ (h^−1^)	κ	zMIC (mg/L)	MIC (mg/L)
Rifampin	0.0453±0.0018	−0.125±0.0072	0.925±0.17	1.27±0.22	0.512
Amikacin	0.0457±0.0012	−0.145±0.0019	1.23±0.12	0.38±0.029	0.5
Clarithromycin	0.0483±0.00068	−0.0434±0.0013	0.783±0.077	1.58±0.14	1
Streptomycin	0.0465±0.0021	−0.134±0.013	0.508±0.11	2.31±0.50	2
Moxifloxacin	0.0478±0.0015	−0.166±0.0052	0.863±0.091	0.461±0.055	0.37

### Two-drug pharmacodynamics

With the PD function parameter estimates for single antibiotics in hand, we proceeded to assess the validity of the two-drug pharmacodynamic function (Equation 3). To accomplish this, we exposed *M. marinum* to combinations of antibiotics, each of which was at some multiple of its respective MIC, and estimated the growth/death rates of the bacteria over 72 hours. Using the differential equation (Equation 4), the estimated single-drug Hill function parameters and different values of α, we compared the observed growth/death rates to those anticipated from the unique α model.

**Figure 2:**
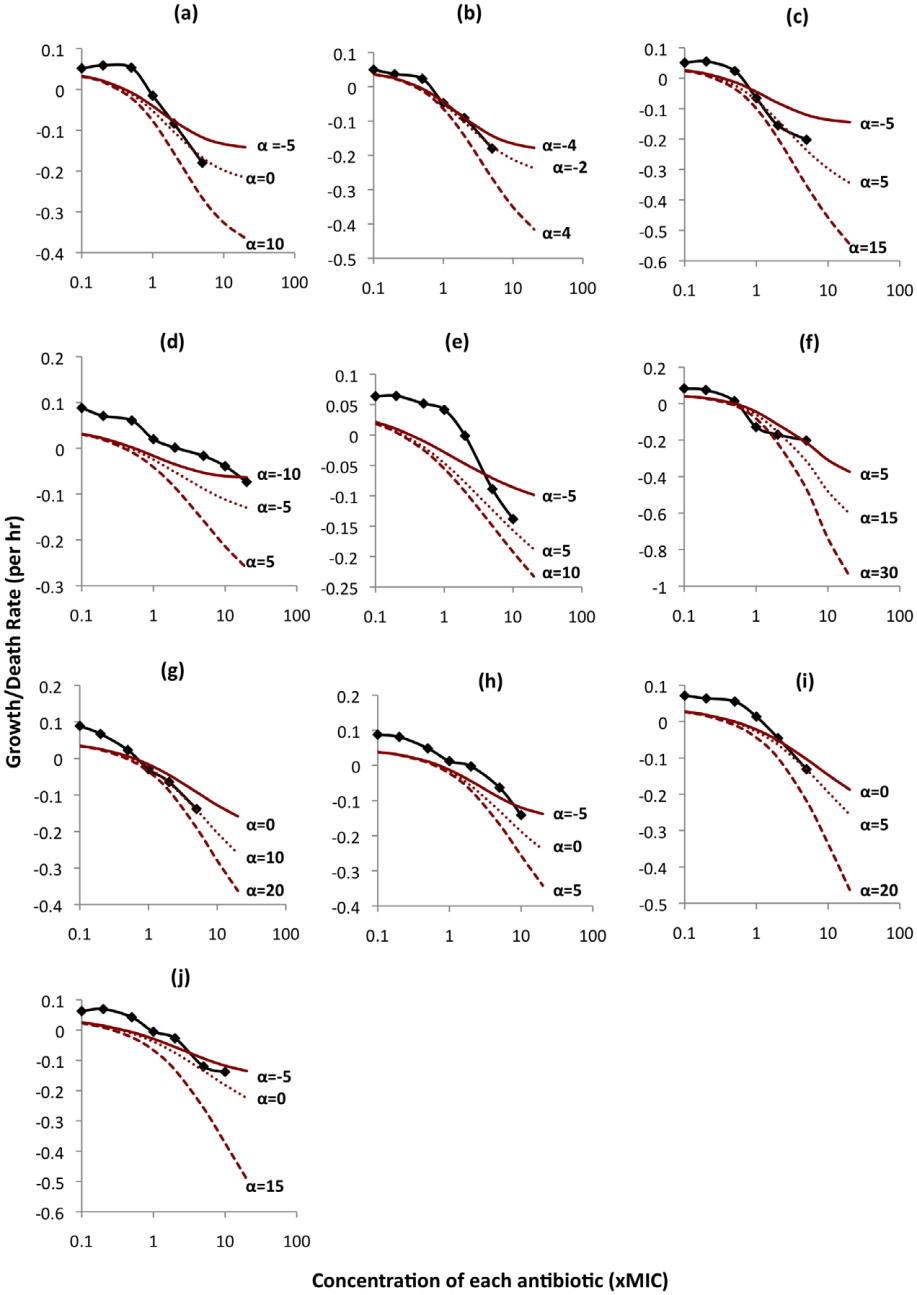
Predicted and observed growth/death rates of *M. marinum* exposed to different combinations of two antibiotics. Curves without markers represent predicted theoretical rates, and curves with markers represent observed experimental rates. Values of α represent different degrees of interaction between antibiotics. Positive values indicate synergy, negative values antagonism, and values of zero, additivity. (a) amikacin + clarithromycin (b) amikacin + moxifloxacin (c) amikacin + streptomycin (d) clarithromycin + moxifloxacin (e) clarithromycin + streptomycin (f) rifampin + amikacin (g) rifampin + clarithromycin (h) rifampin + moxifloxacin (i) rifampin + streptomycin (j) streptomycin + moxifloxacin.

In Figure 2 we show the experimentally-observed changes in bacterial growth/death rates generated by different two-antibiotic combinations (curves with markers) together with those predicted from our model for different drug interaction parameters, the α's (curves without markers). Our estimates of these growth/death rates were limited to situations where the density of surviving cells exceeded 10 CFU per ml. Both the experimental and theoretical analyses were conducted for all possible two-drug combinations of the antimycobacterial drugs used in the study.

For all the drug combinations, it is apparent that a single interaction parameter is insufficient to describe the dynamics over the entire range of concentrations assessed. While the deviation of fit from this single α function varies among antibiotic pairs, in all cases, at lower drug concentrations the observed growth rate is greater than that anticipated from the model. The fit with a single value of α does, however, get somewhat better at higher drug concentrations.

**Figure 3:**
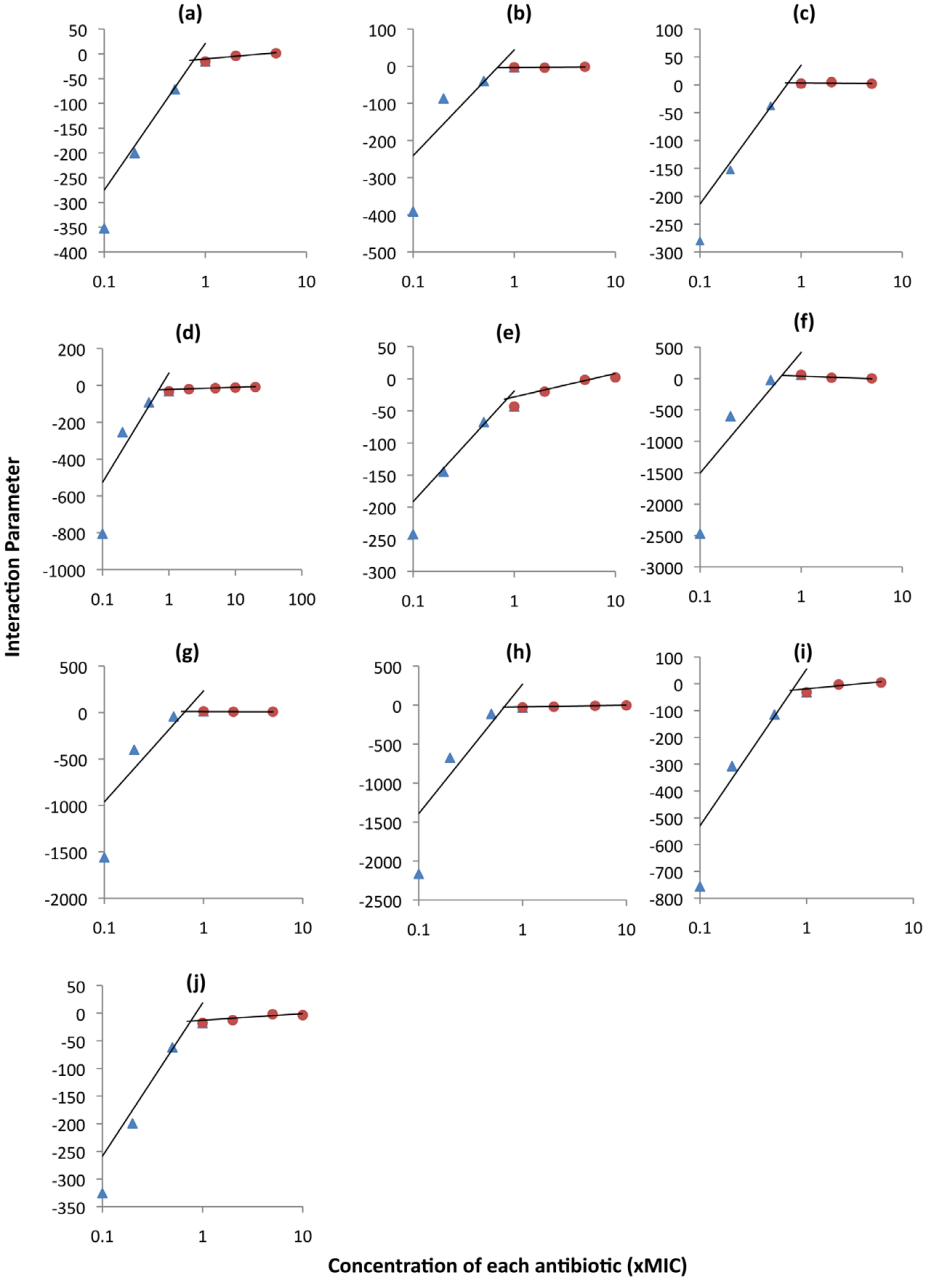
The interaction parameter as a function of antibiotic concentration. Independent linear regressions are shown for sub-MIC (triangles) and supra-MIC (circles) concentrations. (a) amikacin + clarithromycin (b) amikacin + moxifloxacin (c) amikacin + streptomycin (d) clarithromycin + moxifloxacin (e) clarithromycin + streptomycin (f) rifampin + amikacin (g) rifampin + clarithromycin (h) rifampin + moxifloxacin (i) rifampin + streptomycin (j) streptomycin + moxifloxacin.

To get a better idea of the relationship between antibiotic concentration and α, we used Equation 5 to separately estimate this interaction parameter for different concentrations of the ten drug pairs (Figure 3). For all antibiotic combinations, this interaction became relatively more synergistic with increasing drug concentration. Interactions at sub-MIC concentrations were universally antagonistic, but could be mildly antagonistic, additive or synergistic at supra-MIC concentrations (Figure 3 and Table S1). In addition, the rate of change in α from one concentration to the next was much greater at sub-MIC than at supra-MIC concentrations. Interaction coefficients at the larger concentrations only changed to a limited extent and appeared to approach constancy, mirroring the results shown in Figure 2. Although not providing a precise fit to these data, if we assume a two-phase interaction function, one for sub- and one for supra-MIC concentrations and use linear regressions to generate the α functions for each phase, a reasonable fit obtains (Figure 3 and Table S1).

### Asymmetric antibiotic concentrations

For convenience, but also to make this approach to evaluating the pharmaco- and population dynamics of two-drug antibiotic treatment readily applicable, we restricted the above PD experiments to situations in which both antibiotics were at the same xMIC concentration. In an effort to explore the robustness of the two-drug PD observed for these cases of symmetric drug concentrations, we performed time kill experiments for three asymmetric (unequal xMIC concentrations) situations: (i) where both antibiotics are below their respective MICs, (ii) where one antibiotic is below its MIC and the other above and (iii) where both are above their MICs.

When both antibiotics are below the MIC, there is antagonism similar to that observed for the symmetric case. This can be seen in Figure S1, where we present the observed growth rates and those anticipated for situations where there is no interaction between the drugs, α = 0. As would have been anticipated from the symmetric combination results (Figure 2), at sub MICs the drugs together kill at a lower rate than expected were there no interactions between them i.e. they exhibit antagonism. Moreover, the estimated α's for the combination of 0.1 and 0.5 xMIC concentrations of the antibiotics were generally less negative than those calculated for combinations of 0.1-0.1xMIC but more negative than those calculated for the 0.5-0.5 xMIC symmetric cases (Table S2).

Of particular concern in situations where one drug is below the MIC and the other above is that the substantial antagonism observed for below-MIC antibiotic concentrations would be manifest by sub-MIC drugs reducing the efficacy of supra-MIC antibiotics. The results of our experiments indicate that this is not the case (Figure S2). When combined with a sub-MIC concentration of a second drug, the rate of kill of the supra-MIC drug is no less than that when it is alone and in some cases greater.

To explore the effects of asymmetric concentrations for pairs of above-MIC antibiotics, we compared the observed death rate with that anticipated for no interaction between the antibiotics. The results of these experiments suggest that there is either no interaction between the antibiotic pairs or there is the mild antagonism or synergy observed for the symmetric drug concentration experiments (Figure S3).

In sum, the results of these experiments with asymmetric drug concentrations are consistent with that anticipated from the symmetric concentration experiments depicted in Figure 3.

### Predicted dynamics of treatment

**Figure 4:**
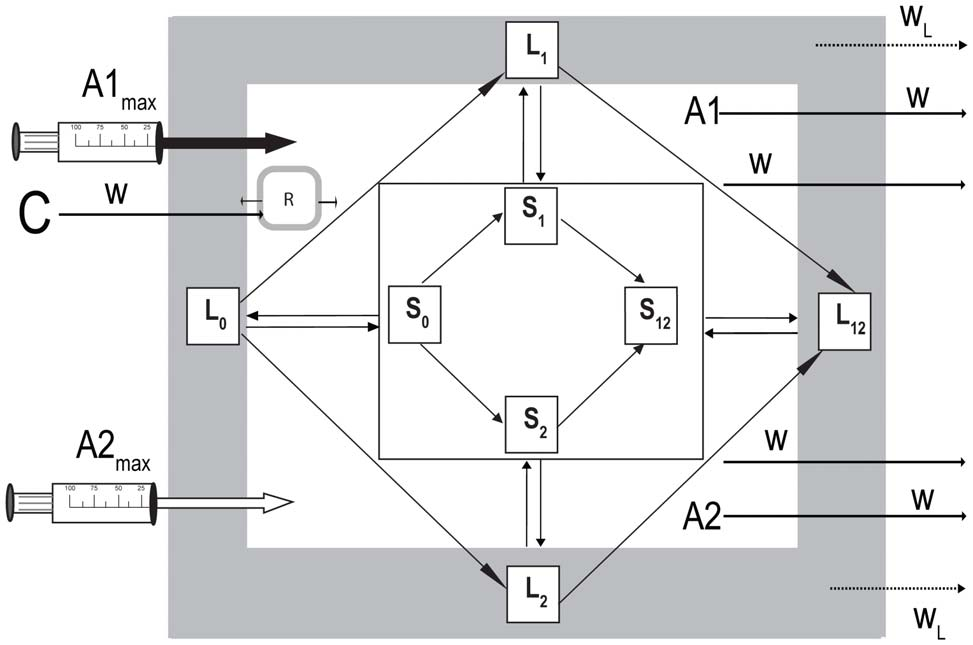
Two-compartment population and evolutionary dynamic model of two-drug antibiotic therapy. Main (active) compartment: S0, bacteria susceptible to both antibiotics; S1, bacteria resistant to antibiotic 1; S2, bacteria resistant to antibiotic 2, S12, bacteria resistant to both antibiotics. Latent (refractory) compartment: L0, bacteria susceptible to both antibiotics; L1, bacteria resistant to antibiotic 1; L2, bacteria resistant to antibiotic 2, L12, bacteria resistant to both antibiotics. C, reservoir resource concentration; R, internal concentration of the limiting resource; A1 and A2, internal concentrations of the antibiotics; A1max and A2max, concentration of antibiotics added periodically; w, flow rate of resources into and out of the compartments; w_L_, flow rate of latent population from the latent compartment.

To evaluate how the pharmacodynamics estimated above would be manifest in a treatment regime, we use a simulation of the within-host population dynamics of bacteria in a two-drug therapy regime for tuberculosis. In Figure 4, we present a diagram of the model used for the analysis (equations for the model can be found in Protocol S1). In designing this model and in choosing the dosing parameters, bacterial densities and PD parameters, we tried to mimic that which would be appropriate for mycobacterial chemotherapy. The structure of our model is based on that suggested by D. Mitchison [38]. It assumes two compartments, one in which the bacteria are actively proliferating and the other where they are dividing slowly and thereby responding differently to antibiotics [39], [40]. This compartment difference in antibiotic susceptibility is reflected in the pharmacodynamic Hill functions, such that the maximum and minimum rates of growth/death are proportional to the rate of replication in the two compartments. The idea is that the slowly dividing subpopulation is relatively refractory to killing by the antibiotics, as would be the case for latent or persister cells in a tuberculosis infection.

We allow for four states of the bacteria, one that is susceptible to both drugs, S0 and L0 (S and L for rapidly- and slowly-dividing populations respectively), S1 and L1 for those resistant to drug 1, S2 and L2 for cells resistant to drug 2, and S12 and L12 for cells that are resistant to both drugs. These variables are both the densities (cells/ml) of bacteria in these states as well as their state designations. By resistance we are assuming that these bacteria are totally refractory to the drugs, with MICs at least 100X that of the susceptible cells. Resistance also engenders a 5% fitness cost which is manifest as a 5% lower maximal growth rate of bacteria in those states. This assumed cost is in the range of what has been observed for *M. marinum* mutants resistant to the antibiotics considered in this study (unpublished results). We allow migration at rates *f_ls_* (from latent to susceptible) and *f_sl_* (from susceptible to latent) cells per hour, representing either a physical or a physiological translocation between the compartments.

Resources for bacterial growth enter and are removed from the habitat (host) at a constant rate, w ml per hour. The bacteria, however, are removed from the habitat at two rates, w for S0, S1, S2 and S12, and w_L_ for L0, L1, L2, and L12, where w> w_L_. For the pharmacodynamic functions, we use the two-drug Hill functions with the biphasic model for the interaction coefficient described above. For pharmacokinetics we assume that a fixed dose A1max and A2max of each drug is added every T hours. In addition to washout at rate w, both drugs also decay at a rate d mg/L per hour. In these simulations we assume that at the onset of treatment, the sensitive population is initially at a density of S0 = 5×10^7^ in the main compartment [41] and L0 = 5×10^4^ cells per ml in the refractory compartment.

As would be anticipated for hosts infected with numbers of bacteria that exceed the reciprocal of the mutation rates, we assume that there are minority populations of bacteria resistant to single antibiotics, S1, S2, L1 and L2, with a relative frequency of 10^−3^ to the corresponding susceptible population [42]. We also allow resistance to single drugs to evolve during the course of the simulations at rates proportional to the product of the number of individuals of each ancestral state and a mutation rate. The actual generation of mutants occurs in a semi-stochastic manner, via a Monte Carlo routine. At each time step (Δt) in the finite step size (Euler) simulation, the probability that a mutant would be generated is the product of the number of individuals of the genotype, Δt and the mutation rate µ. When the random number is less than this product, a mutant is added to the noted population, e.g. when S1 is generated from S0, a bacterium is added to the S1 state and one removed from the S0 state. We use step sizes of Δt so that the probability of a mutant being added at a particular time interval is always less than 1. For these simulations, µ takes values in the range of that estimated from fluctuation experiments for different antibiotics and *M. marinum* (unpublished results). There are no doubly resistant cells, S12 and L12 at the start of the simulations, but they can evolve by mutation from the single resistant states.

**Figure 5:**
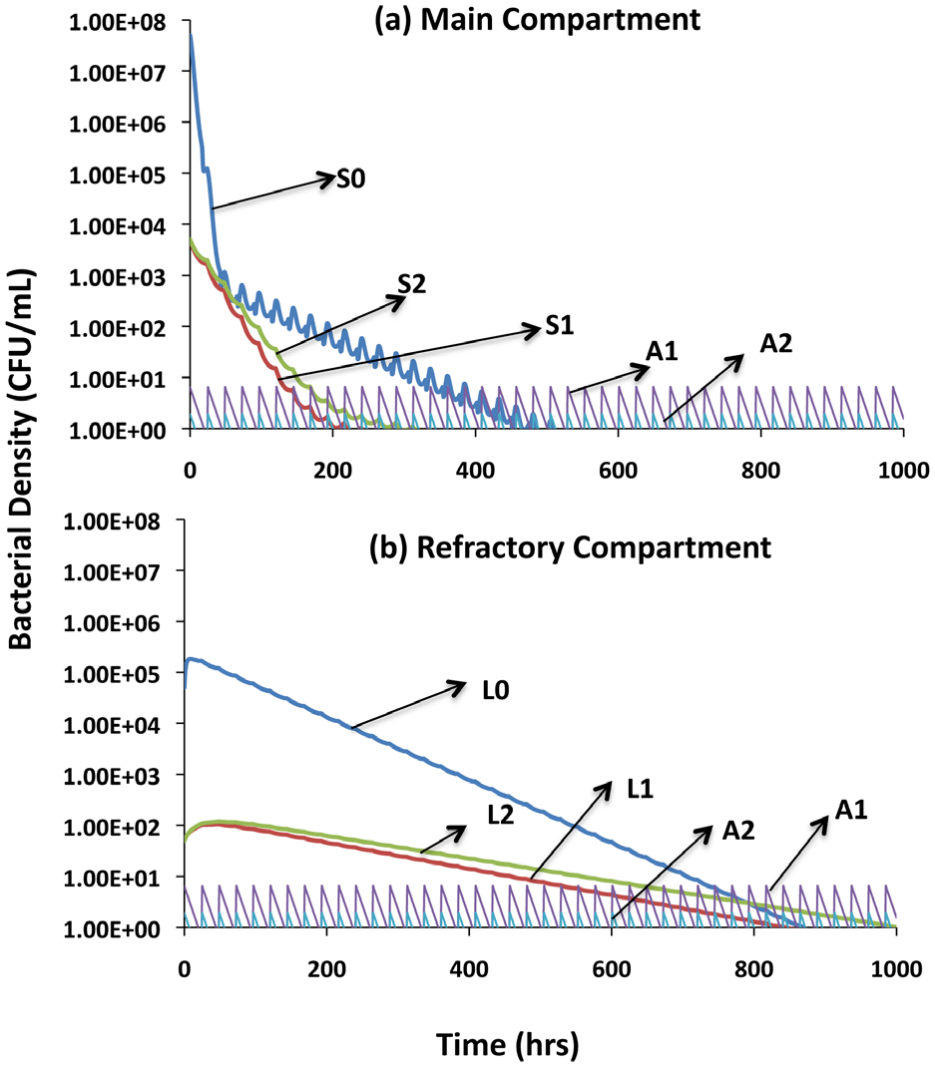
Clearance dynamics for different subpopulations in the main and refractory compartments of the PD/PK model. Parameters used are those observed for the rifampin (A1) + amikacin (A2) combination. In these simulations, w and w_L_ are, respectively, 0.02 and 0.002 per hour; f_ls_ = f_sl_ = 0.001; the antibiotic decay rate is d = 0.075 hr^−1^ and the maximum and minimum bacterial growth rate for each subpopulation in the latent compartment is 10% of those in the active. (a) Main compartment, (b) Refractory compartment.

In Figure 5, we follow the changes in density of the different bacterial populations in the main compartment (5a) and in the refractory compartment (5b). The PD parameter values used in this simulation are those in the range estimated in our experiments for the combination of rifampin (A1) and amikacin (A2). These antibiotics are inoculated every 24 hours at a concentration of 5X their respective MICs and decline in concentration due to flow and a decay rate, d = 0.075 per hour. With these parameters, the overall densities of the sensitive and single-resistant populations continue to decline during the course of the simulation. In the main compartment this decline is punctuated by oscillations in density reflecting the waxing and waning of the antibiotic concentration, with net decline each hour. The single resistant populations are cleared earlier than the sensitive for two reasons: their lower initial densities and their lower fitness relative to the sensitive bacteria. This interpretation was confirmed by running simulations in which single resistant populations were at higher initial densities and had lower fitness costs (data not shown). Under these conditions, their resistance to single antibiotics does not make up for this fitness cost.

In the refractory compartment, the rate of change in cell density is lower and the oscillations are not manifest to the same extent as in the main compartment. This occurs because the replication and washout rates are lower, as is the rate of kill by the antibiotics. As a result of continuous migration of cells from and to the slower-growing population, the rate of decline in the density of cells in the main compartment is reduced whilst that in the refractory compartment increased relative to what would obtain were they the sole compartments or not connected. Said another way, the existence of a refractory compartment prolongs the term of therapy.

To compare the relative efficacy of different combinations of antibiotics, we ran these simulations with the estimated PD parameter values obtained for the different combinations of drugs. In addition to simulations with symmetric antibiotic concentrations for the two drugs, we also conducted these simulation experiments with asymmetric antibiotic concentrations. The former were initiated with 5xMIC of both drugs and the latter with 5xMIC of one antibiotic and 2xMIC of the other. As a result of flow and decay, the asymmetric drug concentration simulations include periods where both drugs are above the MIC, one above and one below, and both below. The interaction coefficients used in these simulations are those estimated from the corresponding symmetric and asymmetric concentration experiments. As our measure of the efficacy of treatment, we considered the time until the total density of bacteria was less than one (time to clearance). The results of these simulations are presented in Table 2. While in some runs doubly resistant mutants emerged, ascended and thereby precluded clearance, these were not included in the Table 2 clearance data. The frequencies of runs in which double resistance emerged are considered separately.

**Table 2:** Relative efficacy of antibiotic combinations in clearing bacteria during simulated infections.

	Time to clearance (hours)	
Antibiotic combination	Antibiotics at symmetric xMIC concentrations	Antibiotics at asymmetric xMIC concentrations
Rifampin + Amikacin	1080	2785
Rifampin + Clarithromycin	1527	2521
Rifampin + Streptomycin	1433	2396
Rifampin + Moxifloxacin	1453	2642
Amikacin + Clarithromycin	1428	2568
Amikacin + Streptomycin	1315	2452
Amikacin + Moxifloxacin	1090	2690
Clarithromycin + Streptomycin	11668	13035
Clarithromycin + Moxifloxacin	4530	5793
Streptomycin + Moxifloxacin	1422	2257

Although mutation is a stochastic process, there was effectively no between-run variation in the time before clearance. For eight out of the ten combinations, clearance occurred in less than 1600 hours. The rifampin + amikacin combination was the most effective, leading to clearance in 1080 hrs. The combinations of clarithromycin + moxifloxacin and clarithromycin + streptomycin took substantially longer to clear the bacteria; compared to the rifampin + amikacin combination, the clarithromycin + moxifloxacin combination took some 4 times longer, with the clarithromycin + streptomycin combination taking approximately 11 times longer. This is what would be anticipated from the relative pharmacodynamics of the different drug combinations (Figure 2).

As in the symmetric case, the majority of the antibiotic combinations in the asymmetric simulations cleared the infection over a relatively similar period, i.e. <2800 hours. The reason that the average time to clearance is greater for the asymmetric concentrations is because there is a lower peak concentration for one of the two drugs, rather than equal peaks. While clarithromycin + streptomycin and clarithromycin + moxifloxacin remained the least effective drugs, the most effective combination was streptomycin + moxifloxacin rather than rifampin + amikacin. Compared to streptomycin + moxifloxacin, clarithromycin + moxifloxacin and clarithromycin + streptomycin took, respectively, approximately 2.5 and 6 times longer to clear the infection.

### The evolution of multiple resistance

What is the relationship between the PD of the antibiotics and the likelihood of mutants resistant to both drugs emerging? To address this question, we separately performed 1000 simulation experiments using three sets of parameters reflecting the ‘extreme’ conditions of relative efficacy for the symmetric combinations: rifampin + amikacin, clarithromycin + moxifloxacin and clarithromycin + streptomycin. The aggregate results from these simulation experiments are presented in column one of Table 3.

**Table 3:** Percent of 1000 runs in which multi-drug resistant mutants emerged by 1000 hours.

		Random non-adherence			
Antibiotic combination	Complete adherence	10% non-adherence	20% non-adherence	Thermostat non-adherence	Extended drug holiday non-adherence
Rifampin + Amikacin	0.8	1.2	1.4	100	1.7
Clarithromycin + Moxifloxacin	1.2	2.1	3.9	1.3	5.2
Clarithromycin + Streptomycin	1.3	2.6	4.1	1.7	5.8

As can be seen, the two-drug resistant population emerged in only a few runs. Although the relative number of runs in which resistance emerged for the different drug combinations is what would be anticipated from the clearance data in Table 2, the differences were not statistically significant (p∼0.525). With these parameters, the frequency of two-drug resistance emerging was low and was roughly the same for all three pairs of drugs.

### Non-adherence

In a number of epidemiological studies, non-adherence to the prescribed treatment regime has been associated with adverse therapeutic outcomes [43], longer terms of treatment and acquired drug resistance [44], [45]. In practice, non-adherence takes a number of forms and depends on a variety of factors such as organization of treatment and care (access to services, length, drug-type and other requirements for therapy, support services, etc) individual interpretations of illness and wellness, drug side effects and the social context in which therapy is undertaken [46]. How does non-adherence contribute to the amount of time required for microbiological cure and the likelihood of multi-drug resistance emerging within a host during the course of treatment? How sensitive are different drug combinations to the adverse outcomes of non-adherence? To address these questions, we considered three broadly-inclusive types of non-adherence that we call random, thermostat [39], and drug holiday (described below). To explore the relationship between the PD of the drug combinations and the frequency of non-adherence with respect to the generation of the double resistant mutants, we conducted 1000 runs for each of the three aforementioned drug combinations and the different non-adherence scenarios. The results of these simulations are presented in Table 3.

### Random non-adherence

**Figure 6:**
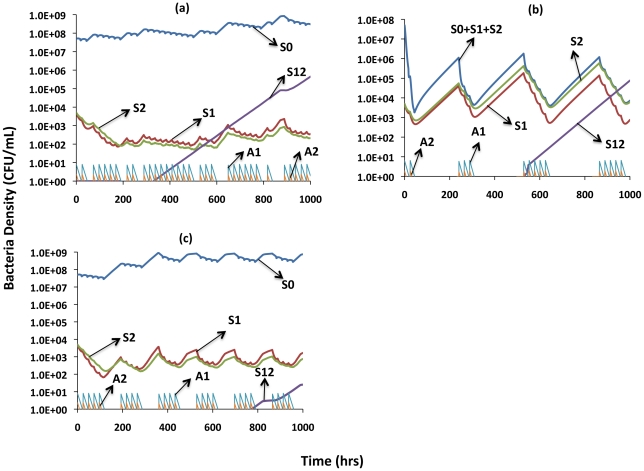
Dynamics of non-adherence with therapy. Changes in the absolute concentrations of the antibiotics and densities of bacteria: S0- sensitive to both drugs, S1- resistant to drug A1, S2- resistant to drug A2, and S12- resistant to both A1 and A2. (a) Random non-adherence: Parameters used are those estimated for clarithromycin + streptomycin, assuming a 20% probability of non-adherence at each dosing. (b) Thermostat non-adherence: Parameters used are those estimated for rifampin + amikacin. (c) Drug holiday non-adherence: Parameters used are those estimated for clarithromycin + moxifloxacin. These figures represent runs in which double resistance (S12) emerged. The relative frequencies of this outcome are shown in Table 3. See the text for descriptions of these different modes of non-adherence.

We model this scenario in the following manner: At each dosing period there is a probability P (0≤P≤1) that both drugs will be taken and a corresponding probability (1-P) that neither will be taken. To simulate this we use a Monte Carlo routine where if the random number, r≤P, the drugs are administered, but if r>P that dosing period is skipped. In Figure 6(a), we illustrate this process for a single run where two-drug resistance emerges. Non-adherence is reflected in a hiatus in the dosing and a rise in the density of all the bacterial populations. There are periods, such as between 600 and 648 hours, where consecutive doses are missed. This results in a substantial rise in the density of bacteria and thereby an increase in the likelihood of a doubly resistant mutant being generated.

With 10% random non-adherence (P = 0.9), there was no significant difference among drug combinations in the probability of resistance arising (p∼0.073) (Table 3, Column 2). With 20% random non-adherence (P = 0.8) there was a highly significant drug combination effect, p∼0.001 (Table 3, Column 3). The likelihood of multiple resistance arising with 20% non-adherence was negatively related to the microbiological efficacy of these different drug combinations. The relationship between the probability of a doubly resistant population emerging for different levels of random non-adherence was also directly related to the microbiological efficacy of the drug combinations. For the rifampin + amikacin combination, there was no significant difference among the 0, 10% and 20% non-adherence regimes (p∼0.435). For the other two pairs, there were significant p<0.001 relationships between the frequency of non-adherence and the likelihood of double resistance emerging.

### Thermostat non-adherence

We simulate this by incorporating a situation in which treatment ceases when the density of the rapidly growing population falls below 10^4^ and doesn't commence again until the density exceeds 10^6^. The situation we are mimicking is one in which patients cease taking their antibiotics when they are feeling better (the bacterial densities are low enough not to be symptomatic) and do not take the drugs again until the density is high enough to be symptomatic. We illustrate this situation in Figure 6(b) with a run in which two-drug resistance emerged.

In column 4 of Table 3, we summarize the results of 1000 simulations of thermostat non-adherence for the three drug combinations. With respect to our measure of microbiological efficacy, the thermostat non-adherence scenario seems paradoxical. Two-drug resistance emerged far more frequently in the runs with the most microbiologically effective drug combination, indeed in all 1000 runs. The reason for this is that the more effective drug combination reduced the density more rapidly than the less effective drug combinations. As a result there were far more frequent periods where drugs were not taken and the single-resistant populations ascended to high-enough densities where two-drug resistant mutants were produced with a very high probability. Under the parameter conditions of this simulation, the non-adherence threshold was never crossed in any of the 1000 simulations for either of the two less effective drugs.

### Drug holidays

We model this scenario in the following manner: Both drugs are taken for 4 consecutive dosing periods, at which time neither drug is taken for the subsequent 3 dosing periods. This regime continues throughout the duration of simulated treatment. We are mimicking a situation where holidays are imposed because the drugs may be costly, limited in their availability or induce debilitating side effects that are alleviated by terminating treatment for an interval. In Figure 6(c) we illustrate this situation for a run where two-drug resistance emerged. As noted in the last column of Table 3, the overall frequency of double resistance was on the order of 5% and similar for the two microbiologically less effective drug combinations. For the most effective drug combination, relative to complete adherence, the drug holidays doubled the likelihood of two-drug resistance emerging.

## Discussion

With few exceptions, studies of the pharmacodynamics (PD) of antibiotics and bacteria have been restricted to single drugs [10]–[19]. Some infections, particularly those that are long-term like tuberculosis, require multiple antibiotics for treatment to be effective. It follows then, that for the rational design of treatment protocols for these infections, multidrug PD analyses are necessary.

Our results indicate that Hill functions provide an excellent fit for the single-drug PD for *Mycobacteria marinum* and each of the five antibiotics considered in this study, amikacin, clarithromycin, moxifloxacin, rifampin and streptomycin. On the other hand, if, as is assumed in the classical model of Greco and colleagues [25], [26], the interactions between drugs is expressed as a single parameter with a constant value, two-drug Hill function models do not fit the PD observed for any of the 10 pairs of drugs considered. In all cases, at lower antibiotic concentrations the interactions between the drugs is antagonistic; they are less effective together than anticipated from their action alone. As the antibiotic concentrations increase, this drug-drug interaction becomes relatively more synergistic and approaches constancy. To address this phenomenon, we allow for two phases of the drug-drug interaction, one for low (sub-MIC) and one for high (supra-MIC) concentrations with an antibiotic concentration-dependent function for the interaction term. Albeit not as convenient as a unique parameter, these functions can be readily estimated from time-kill data. Most importantly, the biphasic drug interaction Hill function models thus generated provide quantitatively accurate analogues of the PDs of all 10 pairs of antibiotics examined.

It has been hypothesized that there are subpopulations of bacteria within an infected TB host that exhibit differential growth rates and, by extension, variable susceptibility to antimycobacterial agents [38], . Here, we develop a simple mathematical model that accounts for this within-host bacterial heterogeneity by assuming that there are two ‘compartments’, one that houses rapidly-growing and the other slowly-growing bacteria. The model incorporates the possibility of non-adherence to therapy, which is considered to be one of the major contributory factors to TB treatment failure [43], [45], [51].

Our computer simulations of tuberculosis chemotherapy employing the empirically estimated biphasic Hill functions suggest that there can be substantial differences among drug combinations in treatment efficacy, as measured by the time to clearance. Of the ten antibiotic pairs we consider, rifampin + amikacin is the most effective and streptomycin + clarithromycin the least, with some eleven-fold difference in the time before clearance. With the parameters used in our semi-stochastic model of treatment and assuming different probabilities for the occurrence of random non-adherence, either complete adherence or limited non-adherence to the therapeutic regime would not be manifest as a significant difference among drug combinations in the likelihood of the generation and ascent of two-drug resistant mutants. However, with greater rates of non-adherence, the likelihood of two-drug resistance emerging becomes increasingly dependent on the drug combination employed. The emergence of two-drug resistance due to random non-adherence is more likely for less microbiologically effective drug combinations than those that are more effective.

With externally imposed regular drug holidays, the likelihood of emergence of two-drug resistance is also inversely proportional to the microbiological efficacy of the antibiotic combination. Our results suggest that quite a different situation obtains when the drug holidays depend on the bacterial load, as is the case for thermostat non-adherence. Under the parameter conditions used in our simulations, the most microbiologically effective drug combination almost invariably leads to the emergence of two-drug resistance. As a result of the enhanced efficacy, the time required to reduce the bacterial densities to below a non-symptomatic threshold is decreased for the more effective antibiotic combination. Consequently, in the course of therapy this threshold and the resulting drug holidays are reached and manifest more frequently for the more effective drug combinations than the less effective. During these holidays, intermediates resistant to single antibiotics can reach high enough densities for the single drug resistant clones to acquire the second mutation needed for two-drug resistance. It is easy to write-off this paradoxical result as an artifact of the model because of the extraordinary frequency of two-drug resistance emerging in our simulations. On the other hand, this outcome is not entirely unreasonable if indeed patients go off treatment when they are no longer symptomatic but remain infected. While we are not championing the validity of this potential downside of effective chemotherapy, we believe it may warrant further consideration.

This jointly theoretical and experimental study raises important as well as intriguing issues about the interactions between antibiotics of different classes and how these interactions are affected by their concentrations. Our results, however, provide no information about the physiological, molecular and other processes underlying these interactions. What are these processes? It is clear that answering this question is not going to be trivial. As Yeh and Kishony argue, intuitive deductions about the type of interactions between drugs based on the metabolic pathways of action of their individual action are, at best, simplifications [52]. Antibiotic action is pleiotropic and not limited to structural or metabolic alterations to a particular target. As such, the resulting cellular death or growth cessation upon antibiotic use can be due to multiple factors. Although there is evidence that antibiotics of different types kill by a common non-specific mechanism, the production of hydroxyl radicals [53]–[55], the rates of kill vary among drugs and their concentrations in ways that cannot be predicted from their respective targets and mode of action.

Particularly intriguing is the antagonistic interaction observed at lower (sub-MIC) concentrations among all the antibiotic pairs studied. Why? How? We know that antibiotics at both sub- and supra-MIC concentrations affect mycobacterial transcription patterns in a variety of ways and can lead to a number of physiological and biochemical stress responses [56], [57]. Some of these responses have been observed to reduce antimicrobial activity through actions such as antibiotic efflux, ribosomal protection, etc [58]–[61]. One possible explanation is that at sub-MIC concentrations for two drugs, these stress responses make the bacteria more refractory to antibiotic activity, but the drugs do not generate enough cidal activity to overcome this refractoriness – a phenomenon that would manifest as pharmacodynamic antagonism.

To paraphrase the statistician George Box, ‘All models (and model systems) are wrong, some are useful’ [62]. We endorse this perspective and of course believe our model and model system are useful. However, we see this utility restricted to its potential to evaluate, *in vitro*, the efficacy of different antibiotic combinations for clinical applications. Our models are not intended to be quantitatively exact analogs of tuberculosis chemotherapy but rather to generate a framework within which questions relevant to TB treatment could be approached. They were designed in the tradition advocated by Richard Levins [63], to maximize reality and generality at the loss of precision. Thus, even though the pharmacodynamic parameters are directly estimated and drug doses simulated in clinically realistic range [64], the time scale in these simulations do not reflect the actual time course of tuberculosis chemotherapy and dosing schedule.

We elected to do the experimental work on this project with *M. marinum* because we are particularly interested in multi-drug treatment of tuberculosis. As a model for Mtb, *M. marinum* has its virtues and limitations. In addition to being more convenient to work with than Mtb, *M. marinum* infections in fish and amphibians demonstrate key elements of Mtb infections in humans [34], [35]. Of particular import is the formation of epitheloid granulomas with lymphocytic involvement [65]. Thus, using either fish or amphibians, it should be possible to evaluate, *in vivo*, the predictions of our models. *M. marinum* is also limited as a model for multi-drug treatment of Mtb primarily because of its natural resistance (relatively high MICs) to some the first line antibiotics used to treat tuberculosis, in particular isoniazid, ethambutol and pyrazinamide. While one of the antibiotics used in this study, rifampin, is a first line tuberculosis drug, the others are only used in cases where first line drugs fail.

Albeit simple, our TB chemotherapy model incorporates some, but clearly not all of the complexity of a *M. tuberculosis* infections and their treatment. It accounts for the subpopulation heterogeneity that has been postulated for these infections [38], and the effects of that heterogeneity on the PD of the antibiotic treatment. On the other hand, this model does not formally account for the third subpopulation suggested by the recent observation that some Mycobacteria in macrophages induce efflux pumps that make them tolerant to antibiotics [66]. At a pharmacodynamic level, this phenomenon is, however, somewhat subsumed in our model by the presence of a subpopulation of bacteria that is less susceptible to the antibiotics than another segment of the population. Additionally, while our model takes into account three forms of the non-adherence that is considered to be one of the major contributory factors to TB treatment failure [43], [45], [51], it certainly does not incorporate all of the nuances of non-adherence.

We are unaware of other studies that have combined experimental work on the PD of multiple drugs with a quantitative consideration of the potential clinical implications of these PDs. There have been investigations of the PD of multiple antibiotics that have employed a fitting approach for a quantitative description of the interactions between drugs [32], [67]. Similar to that observed here, some of these studies provide evidence that the interactions between antibiotics can vary with their concentrations [22], [32], [68]. Nevertheless, to our knowledge, this quantitative relationship has not been taken into account in the design of treatment programs; the interactions between different antibiotics are simply described as additive, synergistic or antagonistic, but without consideration of how this relationship changes with antibiotic concentration. The models we develop and the experimental methods we employ in this study can be used for any combinations of bactericidal antibiotics and bacteria that can be grown *in vitro*. Whether the biphasic interaction phenomenon observed with *M. marinum* and the five drugs considered would be manifest with other bacteria and drug combinations remains to be seen.

## Materials and Methods

### Bacteria and media

*Mycobacterium marinum* strain ATCC BAA-535/M was used in all experiments. Bacteria were grown in Middlebrook 7H9 broth (Difco, Detroit, Mich.) supplemented with 0.2% glycerol and 10% albumin-dextrose complex (7H9) at 32°C. Cell densities were estimated by plating on Middlebrook 7H10 agar (Difco) supplemented with 0.5% glycerol and 10% oleic acid-albumin-dextrose complex (7H10) at 32°C.

### Antibiotics

Rifampin, amikacin, clarithromycin, streptomycin (Sigma, St. Louis, MO, USA) and moxifloxacin (Bayer, Pittsburgh, PA, USA) were purchased commercially. Stock solutions were prepared by dissolving the antibiotics in sterile water or methanol, and appropriate dilutions were made in 7H9 broth immediately before use.

### Time-kill experiments for generating single-antibiotic Hill functions

Mid-log cultures of *M. marinum* were diluted in fresh medium to obtain a density of approximately 5×10^6^ CFU/mL. 200 µL aliquots of this culture were introduced into wells in a 12-well plate containing 1.8 mL of antibiotic solution. The plates were incubated with shaking at 32°C for 72 h, and samples were taken every 12 h to determine viable CFU's.

### MIC determination

Minimum Inhibitory Concentrations (MICs) were estimated using a broth microdilution procedure similar to that recommended by the CLSI[37] (7H9 was used instead of Mueller-Hinton Broth). Initial inoculating bacterial densities were similar to the densities used to initiate time-kill experiments in order to account for the inoculum effect on MIC demonstrated in Udekwu *et al.* [69].

### Antibiotic-kill experiments for generating two-drug PD functions

Antibiotics were combined to generate solutions that contained 0.1, 0.5, 1.0, 2.0, 5.0 and 10.0 multiples of MIC (xMIC) of each antibiotic. Mid-log cultures of *M. marinum* were diluted in fresh medium to obtain a density of approximately 5×10^6^ CFU/mL. 200 µL aliquots of this culture were introduced into wells in a 12-well plate containing 1.8 mL of antibiotic solution. The plates were incubated with shaking at 32°C for 72 h, and samples were taken at the end of the incubation. The experiment was repeated four times, and gave good quantitative and qualitative replication. We show a representative experiment in the Results section of the manuscript.

### Drug interaction modeling

As in Regoes *et al.*, [70] we assume that for single antibiotics, bacterial net growth in the presence of an antibiotic, ψ(A), is dependent on the growth rate of the bacteria in the absence of antibiotics, ψ_ max_, and the death rate due to the antibiotic. The latter is a Hill function, Η, composed of the following parameters: ψ_ max_; ψ_ min_, the maximum antibiotic-generated bacterial killing; zMIC, the pharmacodynamic MIC; and κ, which describes the sigmoidicity of the Hill function [70]. i.e.:
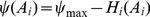
(1)


Where
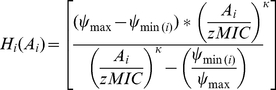
(2)


Bacterial net growth rates were determined from the change in bacterial density over the time-kill period, and the pharmacodynamic function was fit to these data using the least square algorithm nls() of R (www.r-project.org) to obtain estimates for the parameters of the Hill function. For two-antibiotic combinations, we incorporated an interaction parameter (α) into the Hill-function mediated killing by both antibiotics. Thus, net bacterial growth rates would be described by the following equation:
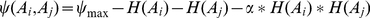
(3)and the rate of change in the viable cell density of bacteria, D, treated with combinations of two drugs given by,
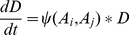
(4)


### Estimation of drug interaction parameter (α)

By assessing bacterial killing over 72 h when exponentially-growing cultures were challenged with pairwise combinations of antibiotics (A_i_ and A_j_) at different concentrations, we obtained empirical estimates for net bacterial growth rates in the presence of both antibiotics, ψ_ exp_. As the theoretical analyses outlined above generate estimates for ψ_max_, H_i_(A_i_) and H_j_(A_j_), algebraically rearranging the net bacterial growth rate equation gives an equation for determining α:
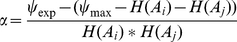
(5)


### Numerical solutions

To follow the predicted change in the viable cell density of bacteria, we use numerical solutions to the differential equation (4) programmed in Berkeley Madonna™. Copies of this program and other programs used in this study and instructions for their use can be obtained on www.eclf.net/programs.

## Supporting Information

Figure S1 **Predicted and observed growth rates of** ***M. marinum*** **exposed to asymmetric sub-MIC antibiotic concentrations.** Blue bars represent predicted rates anticipated from the Hill functions under the assumption that the drugs are acting additively. Red bars represent the growth rates observed for the noted concentrations. Multiples-of-MIC concentrations at which antibiotics are combined are indicated. R-rifampin; A-amikacin; C-clarithromycin; S-streptomycin; M-moxifloxacin. (a) amikacin + clarithromycin (b) amikacin + moxifloxacin (c) amikacin + streptomycin (d) clarithromycin + moxifloxacin (e) clarithromycin + streptomycin (f) rifampin + amikacin (g) rifampin + clarithromycin (h) rifampin + moxifloxacin (i) rifampin + streptomycin (j) streptomycin + moxifloxacin.
(TIF)

Figure S2 **Predicted and observed growth/death rates of** ***M. marinum*** **exposed to sub- and supra-MIC antibiotic combinations.** Growth/death rates observed for single drugs in comparison to that observed with those drugs in combination with a sub-MIC concentration of second antibiotic. Red bars represent combinations of antibiotics at 2xMIC and 0.1xMIC; blue bars represent combinations of antibiotics at 5xMIC and 0.5xMIC. For amikacin, only the lower (2xMIC+0.1xMIC) concentration results are presented. At the higher concentrations the extent of kill exceeded the limit of detection. R-rifampin; A-amikacin; C-clarithromycin; S-streptomycin; M-moxifloxacin. (a) rifampin (b) amikacin (c) clarithromycin (d) streptomycin (e) moxifloxacin.
(TIF)

Figure S3 **Predicted and observed death rates of** ***M. marinum*** **exposed to asymmetric supra-MIC antibiotic concentrations.** Blue bars represent predicted rates anticipated from the Hill functions under the assumption that the drugs are acting additively. Red bars represent the growth rates observed for the noted concentrations. Multiples-of-MIC concentrations at which antibiotics are combined are indicated. R-rifampin; A-amikacin; C-clarithromycin; S-streptomycin; M-moxifloxacin. (a) amikacin + clarithromycin (b) amikacin + moxifloxacin (c) amikacin + streptomycin (d) clarithromycin + moxifloxacin (e) clarithromycin + streptomycin (f) rifampin + amikacin (g) rifampin + clarithromycin (h) rifampin + moxifloxacin (i) rifampin + streptomycin (j) streptomycin + moxifloxacin.
(TIF)

Table S1 **Linear regression parameters for the biphasic antibiotic interaction function.**
(DOC)

Table S2 **Value of interaction parameter at different combinations of sub-MIC concentrations.**
(DOC)

Protocol S1 **Differential equations used for simulation of the mathematical model.**
(DOC)
