# Supplementary figures and images for: Two-Drug Antimicrobial Chemotherapy: A Mathematical Model and Experiments with Mycobacterium marinum
Source: PLoS Pathog. 2012 Jan 12;8(1):e1002487. doi: 10.1371/journal.ppat.1002487 (PMC3257304; doi:10.1371/journal.ppat.1002487)

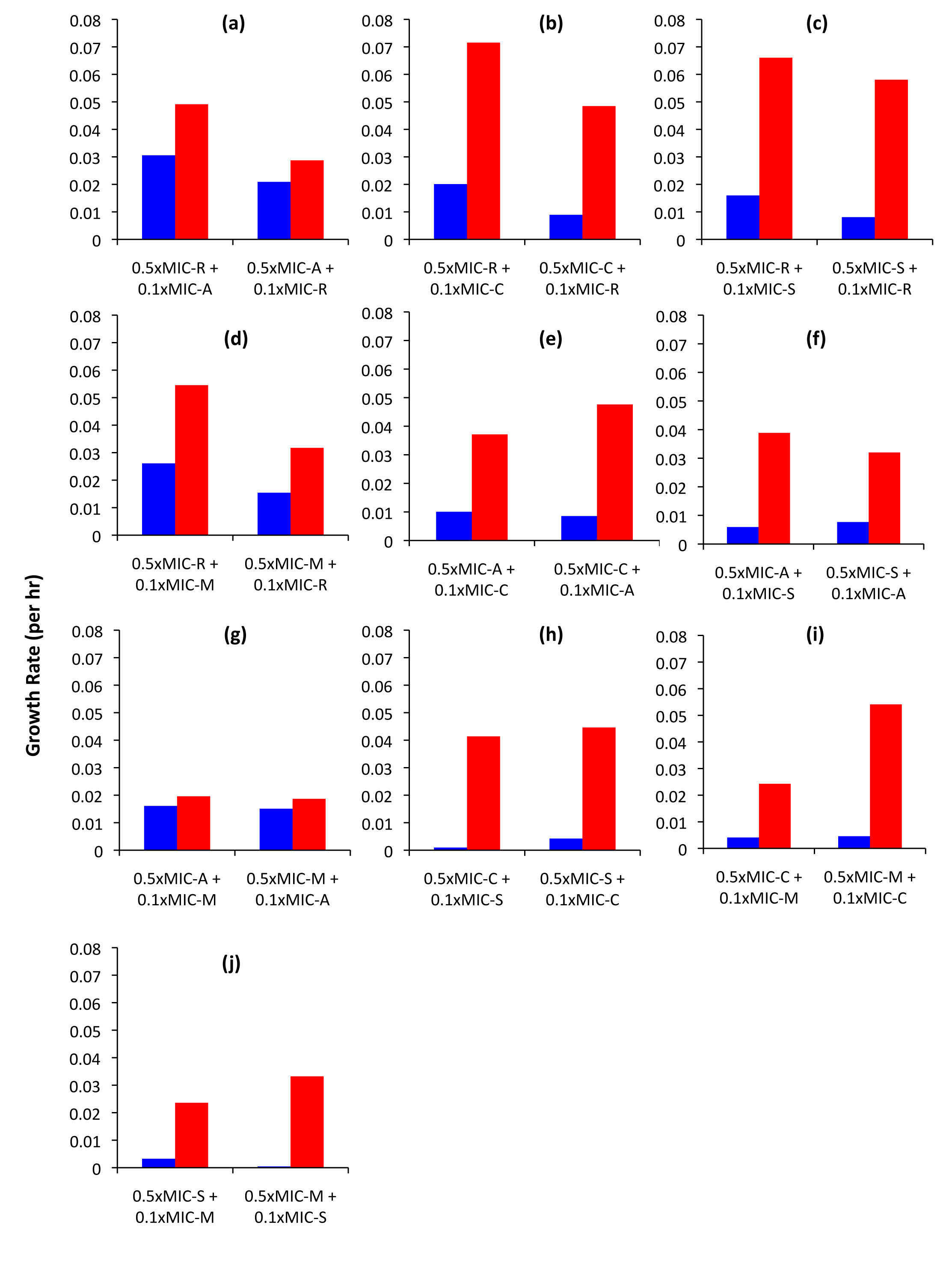

Supplement: Figure S1 — Predicted and observed growth rates of M. marinum exposed to asymmetric sub-MIC antibiotic concentrations. Blue bars represent predicted rates anticipated from the Hill functions under the assumption that the drugs are acting additively. Red bars represent the growth rates observed for the noted concentrations. Multiples-of-MIC concentrations at which antibiotics are combined are indicated. R-rifampin; A-amikacin; C-clarithromycin; S-streptomycin; M-moxifloxacin. (a) amikacin + clarithromycin (b) amikacin + moxifloxacin (c) amikacin + streptomycin (d) clarithromycin + moxifloxacin (e) clarithromycin + streptomycin (f) rifampin + amikacin (g) rifampin + clarithromycin (h) rifampin + moxifloxacin (i) rifampin + streptomycin (j) streptomycin + moxifloxacin. (TIF) [file ppat.1002487.s001.tif]

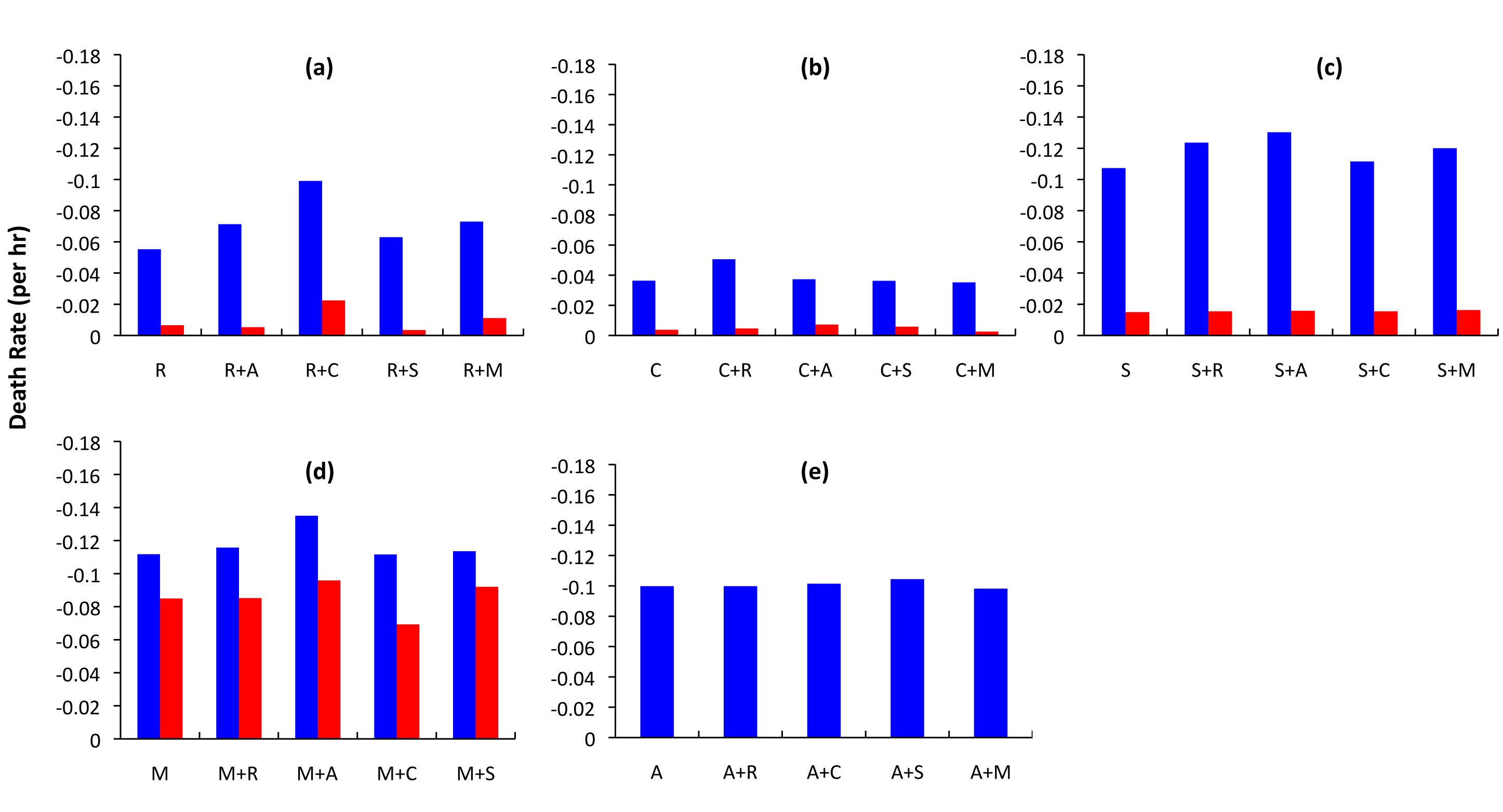

Supplement: Figure S2 — Predicted and observed growth/death rates of M. marinum exposed to sub- and supra-MIC antibiotic combinations. Growth/death rates observed for single drugs in comparison to that observed with those drugs in combination with a sub-MIC concentration of second antibiotic. Red bars represent combinations of antibiotics at 2xMIC and 0.1xMIC; blue bars represent combinations of antibiotics at 5xMIC and 0.5xMIC. For amikacin, only the lower (2xMIC+0.1xMIC) concentration results are presented. At the higher concentrations the extent of kill exceeded the limit of detection. R-rifampin; A-amikacin; C-clarithromycin; S-streptomycin; M-moxifloxacin. (a) rifampin (b) amikacin (c) clarithromycin (d) streptomycin (e) moxifloxacin. (TIF) [file ppat.1002487.s002.tif]

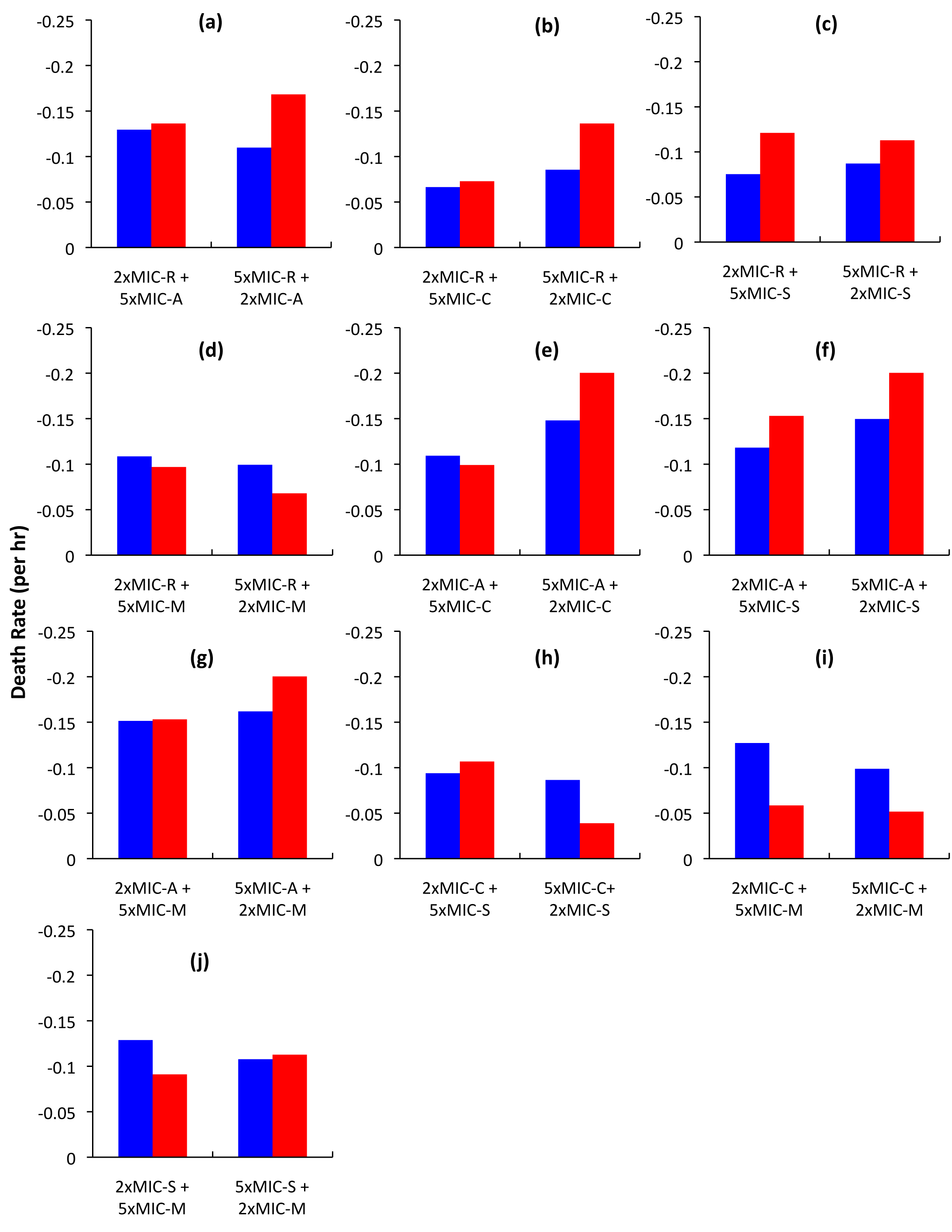

Supplement: Figure S3 — Predicted and observed death rates of M. marinum exposed to asymmetric supra-MIC antibiotic concentrations. Blue bars represent predicted rates anticipated from the Hill functions under the assumption that the drugs are acting additively. Red bars represent the growth rates observed for the noted concentrations. Multiples-of-MIC concentrations at which antibiotics are combined are indicated. R-rifampin; A-amikacin; C-clarithromycin; S-streptomycin; M-moxifloxacin. (a) amikacin + clarithromycin (b) amikacin + moxifloxacin (c) amikacin + streptomycin (d) clarithromycin + moxifloxacin (e) clarithromycin + streptomycin (f) rifampin + amikacin (g) rifampin + clarithromycin (h) rifampin + moxifloxacin (i) rifampin + streptomycin (j) streptomycin + moxifloxacin. (TIF) [file ppat.1002487.s003.tif]
