# Supplementary material for: Two-Drug Antimicrobial Chemotherapy: A Mathematical Model and Experiments with Mycobacterium marinum
Source: PLoS Pathog. 2012 Jan 12;8(1):e1002487. doi: 10.1371/journal.ppat.1002487 (PMC3257304; doi:10.1371/journal.ppat.1002487)
Supplement: Table S1 — Linear regression parameters for the biphasic antibiotic interaction function. (DOC) [file ppat.1002487.s004.doc]

Table S1. Linear Regression Parameters for the Biphasic Antibiotic Interaction Function.

| Antibiotic Combination | Sub-MIC Antibiotic Concentrations | | | Supra-MIC Antibiotic Concentrations | | |
| --- | --- | --- | --- | --- | --- | --- |
|  | Slope | Intercept | R^2^ | Slope | Intercept | R^2^ |
| Amikacin + Clarithromycin | 329.22 | -308.12 | 0.79 | 3.606 | -15.676 | 0.77 |
| Amikacin + Moxifloxacin | 316.26 | -272.49 | 0.52 | 0.4221 | -3.9734 | 0.53 |
| Amikacin + Streptomycin | 276.93 | -241.77 | 0.77 | -0.2866 | 3.8084 | 0.15 |
| Clarithromycin + Moxifloxacin | 659.53 | -592.83 | 0.57 | 0.9247 | -24.867 | 0.64 |
| Clarithromycin + Streptomycin | 191.33 | -210.46 | 0.74 | 4.3471 | -35.223 | 0.71 |
| Rifampin + Amikacin | 2135.8 | -1720.5 | 0.54 | -11.794 | 56.529 | 0.65 |
| Rifampin + Clarithromycin | 1328.8 | -1095.5 | 0.54 | -0.6755 | 12.103 | 0.34 |
| Rifampin + Moxifloxacin | 1845.8 | -1576 | 0.57 | 2.6737 | -27.569 | 0.85 |
| Rifampin + Streptomycin | 649.7 | -594.83 | 0.66 | 7.5399 | -29.97 | 0.67 |
| Streptomycin + Moxifloxacin | 308.13 | -289.7 | 0.79 | 1.4934 | -15.582 | 0.65 |
