# Supplementary material for: Two-Drug Antimicrobial Chemotherapy: A Mathematical Model and Experiments with Mycobacterium marinum
Source: PLoS Pathog. 2012 Jan 12;8(1):e1002487. doi: 10.1371/journal.ppat.1002487 (PMC3257304; doi:10.1371/journal.ppat.1002487)
Supplement: Table S2 — Value of interaction parameter at different combinations of sub-MIC concentrations. (DOC) [file ppat.1002487.s005.doc]

|  | Interaction Parameter | | | |
| --- | --- | --- | --- | --- |
| Antibiotic Combination[[1]](#footnote-2) | 0.1xMIC + 0.1xMIC | 0.1xMIC(Antibiotic A) + 0.5xMIC (Antibiotic B) | 0.5xMIC (Antibiotic A) + 0.1xMIC (Antibiotic B) | 0.5xMIC+0.5xMIC |
| Rifampin + Amikacin | -2470 | -97.1 | -355.8 | -25.7 |
| Rifampin + Clarithromycin | -1559.6 | -326.6 | -259.2 | -42.7 |
| Rifampin + Streptomycin | -756.1 | -403.2 | -195.4 | -115 |
| Rifampin + Moxifloxacin | -2163.9 | -164.5 | -248.1 | -112.5 |
| Amikacin + Clarithromycin | -352.3 | -290.5 | -79.3 | -71.8 |
| Amikacin +Streptomycin | -280.4 | -176.4 | -74.7 | -37.9 |
| Amikacin + Moxifloxacin | -390.9 | -32.5 | -17.9 | -40.0 |
| Clarithromycin + Streptomycin | -242.1 | -77.2 | -61.3 | -67.5 |
| Clarithromycin + Moxifloxacin | -805 | -118.2 | -68.3 | -92.6 |
| Streptomycin + Moxifloxacin | -325.6 | -60.6 | -67.3 | -61.8 |

Table S2. Value of interaction parameter at different combinations of sub-MIC concentrations.

1. First Antibiotic named in the combination is Antibiotic A [↑](#footnote-ref-2)
