# Supplementary material for: Two-Drug Antimicrobial Chemotherapy: A Mathematical Model and Experiments with Mycobacterium marinum
Source: PLoS Pathog. 2012 Jan 12;8(1):e1002487. doi: 10.1371/journal.ppat.1002487 (PMC3257304; doi:10.1371/journal.ppat.1002487)
Supplement: Protocol S1 — Differential equations used for simulation of the mathematical model. (DOC) [file ppat.1002487.s006.doc]

Protocol S1. Differential equations used for simulation of the mathematical model.

$$\begin{matrix} \frac{dS0}{dt}=g_{S0}S0-wS0-2\mu S0-f_{sl}S0+f_{ls}L0 \\ \frac{dS1}{dt}=g_{S1}S1-wS1+\mu S0-\mu S1-f_{sl}S1+f_{ls}L1 \\ \frac{dS2}{dt}=g_{S2}S2-wS2+\mu S0-\mu S2-f_{sl}S2+f_{ls}L2 \\ \frac{dS12}{dt}g_{12}S12-wS12+\mu S1+\mu S2-f_{sl}S12+f_{ls}L12 \\ \frac{dL0}{dt}=g_{L0}L0-w_{L}L0-2\mu L0+f_{sl}S0-f_{ls}L0 \\ \frac{dL1}{dt}=g_{L1}L1-w_{L}L1+\mu L0-\mu L1+f_{sl}S1-f_{ls}L1 \\ \frac{dL2}{dt}=g_{L2}L2-w_{L}L2+\mu L0-\mu L2+f_{sl}S2-f_{ls}L2 \\ \frac{dL12}{dt}=g_{L12}L12-w_{L}L12+\mu L1+\mu L2+f_{sl}S12-f_{ls}L12 \\ \text{where }\mu\text{ per cell per unit time is the mutation rate for change from cells of the } \\ \text{noted to the resistant state, e.g. S0--> S1.} \\ \text{[Growth Rates]} \\ g_{S0}=\psi_{\max}-H(A1)-H(A2)-\alpha(A1,A2)H(A1)H(A2) \\ g_{S1}=\psi_{\max}(1-s_{1})-H(A2) \\ g_{S2}=\psi_{\max}(1-s_{2})-H(A1) \\ g_{S12}=\psi_{\max}(1-s_{12}) \\ g_{L0}=\psi_{\max L}-H(A1)-H(A2)-\alpha(A1,A2)H(A1)H(A2) \\ g_{L1}=\psi_{\max L}(1-s_{1})-H(A2) \\ g_{L2}=\psi_{\max L}(1-s_{2})-H(A1) \\ g_{L12}=\psi_{\max L}(1-s_{12}) \end{matrix}$$

where s_1_, s_2_ and s_12_ (0 ≤ s_x_≤1) are the selection coefficients (fitness costs of resistance) and α(A1,A2) is the antibiotic concentration–dependent drug-drug interaction coefficient (see text).
